# Effects of nutritional and hydration strategies during ultramarathon events between finishers and non-finishers: A systematic review protocol

**DOI:** 10.1371/journal.pone.0272668

**Published:** 2022-08-19

**Authors:** James W. Navalta, Victor D. Y. Beck, Taylor M. Diaz, Vernice E. Ollano

**Affiliations:** 1 Department of Kinesiology and Nutrition Sciences, University of Nevada, Las Vegas, Las Vegas, NV, United States of America; 2 Department of Physical Therapy, University of Nevada, Las Vegas, Las Vegas, NV, United States of America; Universidade Federal de Minas Gerais, BRAZIL

## Abstract

Ultramarathon running is a sport that is growing in popularity. Competing in an ultramarathon event is physiologically taxing on the human body, and it should not be surprising that not all individuals who enroll for an event ultimately finish. While many factors can contribute to this phenomenon, it is likely that nutritional and hydration strategies play a large role between finishing and not finishing an ultramarathon. No published paper has systematically reviewed the effects of nutritional and hydration strategies during ultramarathon events between finishers and non-finishers. This paper details our intended protocol with the following steps that create the flow of the systematic review: 1) Determine the review question and Participant, Intervention, Comparator, Outcome, Study Design (PICOS) criteria; 2) Create inclusion and exclusion criteria; 3) Create and follow a search strategy; 4) Document sources that are included and excluded according to the pre-determined eligibility criteria; 5) Assess final sources for risk of bias; 6) Extract pertinent data from final full-text articles and synthesize the information; and 7) Disseminate findings of the systematic review.

## Introduction

Ultramarathon running is a sport that is growing in popularity. The oldest ultramarathon event, the Comrades Marathon, began in 1921 covering 89.9 km (55.9 mi), and 17 of the 34 participants did not finish (50% DNF) [1]. Since that time, over 300,000 people have completed the race, which currently caps yearly enrollment at 20,000 participants [2]. Participation in ultramarathon events has risen exponentially since the year 2000, with over a million runners participating (1,042,156) [3]. The sport involves running or walking a distance greater than a traditional marathon (42.2 km, or 26.2 mi). The most popular (over three-quarters of a million entrants) ultramarathon distance is 50 km (31.1 mi) as it represents a distance just above the traditional marathon [3]. Participation decreases as the distance gets longer (just over 100,000 entrants for 100km [62.1 mi], and approximately 40,000 for 24 h events) [3].

Competing in an ultramarathon event is physiologically taxing on the human body. Ultramarathon running has been associated with an increase in cardiac troponin T (a measure of myocardial damage) [4], malondialdehyde and creatine kinase (lipid peroxidation) [5], and oxidative stress [6]. Other reported effects include measures associated with cardiac fatigue such as increased atrial volume [7]. Given these effects it should not be surprising that not all individuals who enroll for an ultramarathon event ultimately finish. It is estimated that between 20–50% of individuals who begin an ultramarathon do not finish [8]. While many factors such as gastrointestinal distress and discomfort can contribute to this phenomenon, it is likely that nutritional and hydration strategies play a large role.

Several systematic reviews have been conducted on various ultramarathon running topics including psychology [9], limiting factors [10], long-term health problems [11], and pathophysiology [12]. There has been a 50-year State of the Science offering [13], as well as a Position Statement on Nutrition specific to training for a single-stage ultramarathon [14]. To our knowledge, no published paper has reviewed the effects of nutritional and hydration strategies during ultramarathon events between finishers and non-finishers. A “systematic review” was selected as the methodology after reading published guidance on review types [15, 16]. The primary aim of the proposed systematic review will be to find and describe the nutritional and hydration strategies between single-stage ultramarathon finishers and non-finishers, and outcomes reported in published literature. Based on the Preferred Reporting Items for Systematic Reviews and Meta-Analyses (PRISMA) guidelines, the best practices for performing a systematic review include publishing the protocol independent of the review to ensure procedural transparency [17]. As our systematic review of ultramarathon nutritional strategies will be, to our knowledge, the first published on the topic, it is especially important to publish our protocol. This methods paper was written to achieve two objectives: 1) To adhere to the best practices stated in the PRISMA guidelines; and 2) To ensure procedural transparency.

## Materials and methods

Developing the systematic review protocol began with members of the team performing scoping searches in Google Scholar and PubMed. The scoping searches suggested that there were no published systematic reviews of nutritional strategies between finishers and non-finishers of ultramarathon events. The first author consulted peer-reviewed guidance [15, 16] about conducting systematic reviews in health fields and has participated in the process previously [18]. The team deliberated and agreed upon the protocol presented in this article. The details of the protocol are available via PROSPERO, an online international prospective register of systematic reviews. The protocol was submitted on February 9, 2022 and registered on March 3, 2022 (PROSPERO ID: 42022308733). The protocol has the following steps that create the flow of the systematic review: 1) Determine the review question and Participant, Intervention, Comparator, Outcome, Study Design (PICOS) criteria; 2) Create inclusion and exclusion criteria; 3) Create and follow a search strategy; 4) Document sources that are included and excluded according to the pre-determined eligibility criteria; 5) Assess final sources for risk of bias; 6) Extract pertinent data from final full-text articles and synthesize the information; and 7) Disseminate findings of the systematic review. An optional part of systematic reviews that will be omitted is a meta-analysis. A meta-analysis will not be performed in this systematic review because the studies are expected to include different ultramarathon distances, sample different populations, and measure different outcomes. Because of differences among these characteristics, the studies will not be homogenous, which is necessary for a meta-analysis [19].

## Step 1: Determine the review question and PICOS criteria

The first step is to determine the review question and PICOS criteria (Table 1). Because multi-stage ultramarathon events stress the body from a nutritional and hydrational standpoint in a much different manner compared to single-stage events, we decided to focus on single-stage events not lasting longer than 24 h. The event can be performed in any location (including indoors on a treadmill). Studies with adults who enrolled in a single-stage ultramarathon between the ages of 18–60 will be relevant. Controlled, uncontrolled, randomized, nonrandomized, and observational studies will be considered. Information about participants’ characteristics, ultrarunning experience, and finisher or non-finisher status will be collected.

**Table 1:** Review question and PICOS table.

**Review Question**	Do finishers of single-stage ultramarathon events employ different nutritional and hydration strategies than individuals who do not finish?
**P** **opulation**	Ultramarathoners of any sex who enrolled in a single-stage ultramarathon event, between the ages of 18–60 years old
**I** **ntervention**	Single-stage ultramarathon event not longer than 24 h • The ultramarathon must be performed continuously within 24 h and excludes multi-stage events
**C** **omparator**	Completion of the single-stage ultramarathon event Non completion of the single-stage ultramarathon event (considered as ‘did not finish’ or ‘DNF’)
**O** **utcomes**	Nutrition and hydration strategies employed during the event • Macromolecules (carbohydrates, fats, proteins) • Hydration (fluid type and pacing) • Other supplements (vitamins and minerals) Ultramarathon event characteristics: distance of race, start time, elevation profile, environmental profile (temperature, humidity, windspeed) Participants’ age, sex, body mass, height, years of ultrarunning experience, finisher or non-finisher
**Setting**	Any physical environment (indoors, outdoors, urban, rural, built-up, or natural)
**S** **tudy Design**	Studies with interventions, as well as observational studies • Controlled or uncontrolled • Randomized or nonrandomized

## Step 2: Create inclusion and exclusion criteria

The second step is to create inclusion and exclusion criteria, or eligibility criteria. The review question and PICOS criteria form the basis of this systematic review’s eligibility criteria, which aim to be both explicit and succinct. These characteristics provide clarity to the review team members, enabling the exclusion of irrelevant sources during the screening process. Expeditious screening is important to the process because teams may be required to screen hundreds to thousands of sources. In consultation of the review question and PICOS criteria, the eligibility criteria were created (Table 2). The inclusion criteria will allow for the inclusion of unpublished master’s theses and doctoral dissertations. This decision was made to capture as much data about nutritional and hydrational strategies during a single-stage ultramarathon event as possible.

**Table 2:** Eligibility criteria.

**Participants**	Adults between the age of 18–60 years old, and any sex, gender, or nationality who has enrolled in a single-stage ultramarathon not lasting longer than 24 h
**Inclusion Criteria**	1. The source is a published article in a peer-reviewed journal or is an unpublished or findable master’s thesis or doctoral dissertation 2. The source is written in English 3. The source reports the findings of an interventional or observational study a. The intervention is any nutritional or hydrational supplement b. At least one reported outcome is finishing or not finishing an ultramarathon event c. The observation is nutritional, or hydration strategy utilized while performing an ultramarathon event
**Exclusion Criteria**	1. The source is not a published, peer-reviewed journal article or a findable and available master’s thesis or doctoral dissertation 2. The source is written in any language other than English 3. The source reports the findings of an interventional study with an intervention or outcomes irrelevant to this systematic review a. The intervention is an ultramarathon without nutritional or hydration component b. None of the reported outcomes are finishing or not finishing the ultramarathon event

## Step 3: Create and follow a search strategy

After determining eligibility criteria, the review team will proceed to the third step by creating and following a search strategy (Table 3). Consistent with our PICOS and eligibility criteria, we will search in four databases for all relevant studies, regardless of publication year. The same search combination string will be used in each database (Table 3). The search combination was initially created by the full review team. An iterative process of revision ensued until the search combination returned a manageable number of hits in each database (about 100–1,000 hits).

**Table 3:** Search strategy.

**Investigators**	Team A: TD and JN Team B: VB and VO	
**Techniques**	Search research databases for sources, including them in four stages: 1. Include sources by title 2. Include sources by abstract 3. Include sources by full text 4. Include sources from the reference lists of sources included by full text (journal articles, master’s theses, and doctoral dissertations)	
**Databases**	Google Scholar, PubMed, and SPORTDiscus, Web of Science	
**Included Types of Literature**	Published, peer-reviewed journal articles; unpublished and published master’s theses and doctoral dissertations	
**Publication Date Range**	No limit	
**Intervention Search Terms**	**Outcome Search Terms**	
“Ultramarathon” “24h ultramarathon” “Ultra endurance” “24h race”	“Finish” “Completion” “Complete” “DNF” “Dropout”	“Nutrition” “Carbohydrate” “Fats” “Protein” “Vitamins” “Minerals” “Hydration” “Electrolytes” “Water” “Fluid” “Supplements” “Supplementation”
**Search Combination**	((ultramarathon) OR (“24h ultramarathon”) OR (“ultra endurance”) OR (“24h race”)) AND ((finish) OR (completion) OR (complete) OR (DNF) OR (“drop out”)) AND ((nutrition) OR (carbohydrate) OR (fats) OR (protein) OR (vitamins) OR (minerals) OR (hydration) OR (electrolytes) OR (water) OR (fluid) OR (supplements) OR (supplementation))	

**Fig 1:**
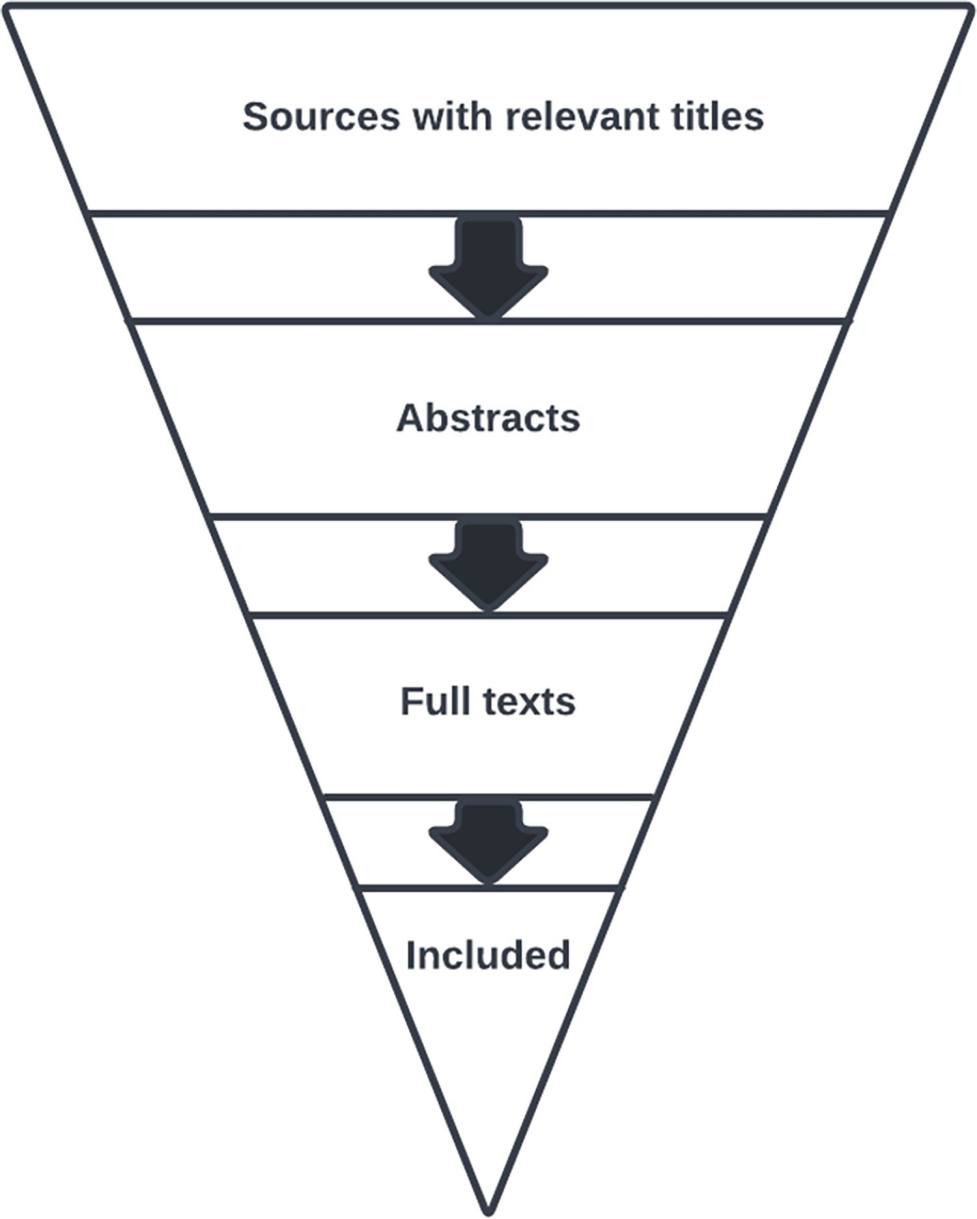
The search flow funnels sources into smaller collections until the final articles are included.

With the operational search combination established, each member volunteered for roles. Two members formed Team A, and two members formed Team B. The two members of Team A will search in Google Scholar and SPORTDiscus. The two members of Team B will search in PubMed and Web of Science. This assignment of databases will divide the workload of the search somewhat equally between the teams. The search, screening process, and inclusion process represents the “search flow.” The search flow will channel an initially broad collection of sources into increasingly smaller collections (Fig 1). The search flow is described in Step 4.

## Step 4: Document sources that are included and excluded according to the pre-determined eligibility criteria

**Fig 2:**
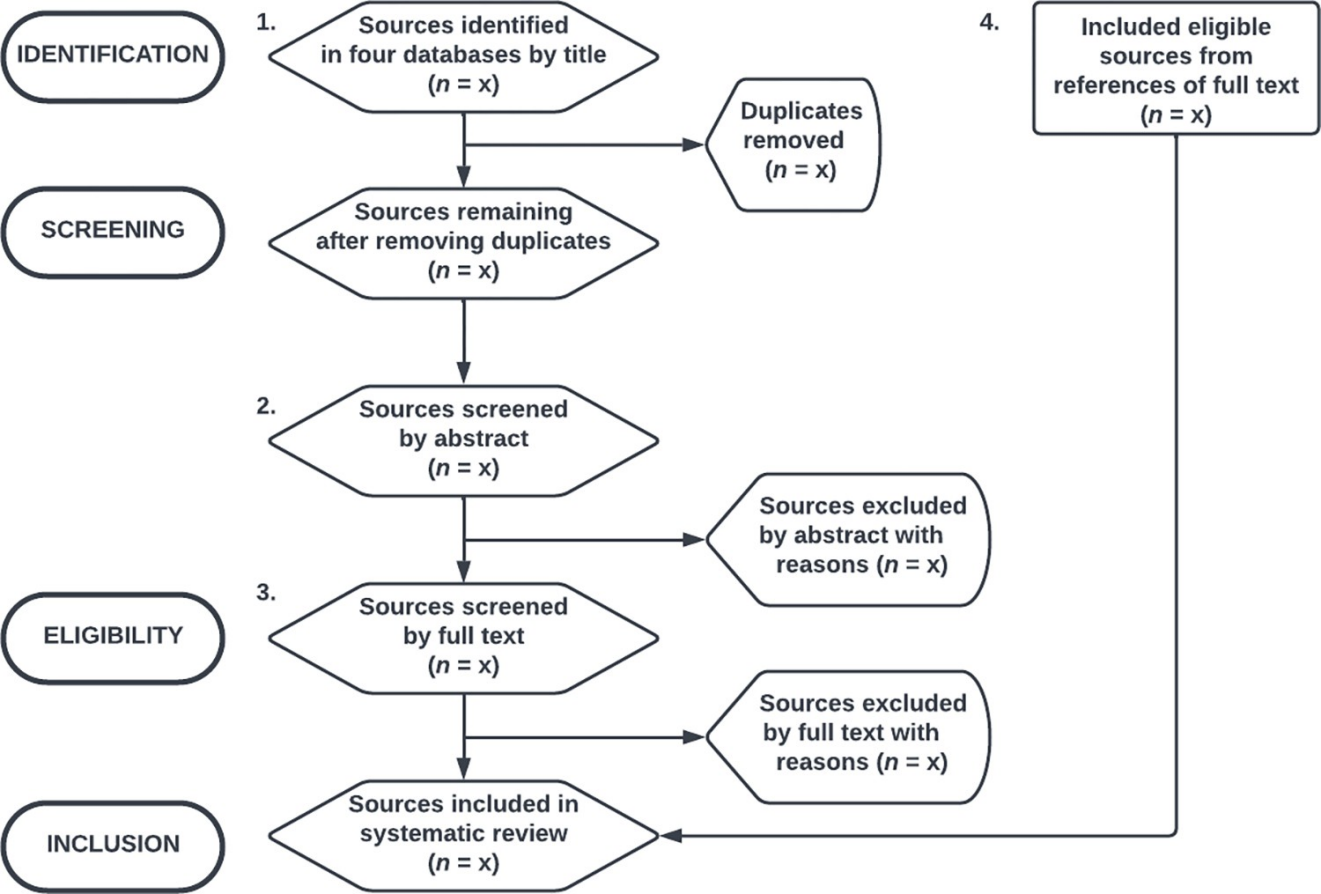
Search flow.

The fourth step is the application of the systematic review process, the search flow (Fig 2). The search flow is modeled on the 2020 PRISMA statement [17], containing four steps: 1) Identify relevant sources by title, 2) Screen sources by abstract, 3) Assess and include sources by full text, and 4) Include eligible sources from the references of full texts included in the third step. During the search flow, it will be critical to document sources’ inclusion and exclusion clearly [16, 19]. Clear documentation allows the systematic review to be transparent and reproducible. Reproducibility is a hallmark of a systematic review that sets it apart from traditional literature reviews [19].

Eligibility criteria will be determined by four individuals (two independent teams of two—Team A and Team B) who will work on selecting the studies. Individuals on the same team will be blinded to the other’s decisions with screening completed independently. The alternate team will resolve any disagreements.

To make the search flow reproducible, a specific tool in Google Sheets will be utilized [20]. The tool is a spreadsheet for Teams A and B to coordinate with each other and has four separate sheets (one for each team member). During the first step of the search flow, members of Team A and B will enter three types of values into the sheet: 1) the number of hits each database returns, 2) the number of sources deemed relevant by title, and 3) the number of duplicate sources identified across the databases (identical sources found in the other database). The sheet will automatically sum these values. The sheet represents a precise record of members’ progression through the first step of the search flow. The sheet also helps members record Steps 2–4.

## Step 5: Assess final sources for risk of bias

The fifth step acknowledges that it is important for all systematic reviews to assess the included sources’ risk of bias [16, 19]. Being transparent about the risk of bias allows readers to draw conclusions concerning the quality and strength of evidence for interventions affecting the outcome [16]. This systematic review will assess risk of bias at the study-level using tools specific to the study design. Randomized, parallel trials will be assessed by using the revised Cochrane risk of bias tool for randomized trials (RoB 2) [21]. Non-randomized trials will be assessed by using the risk of bias in non-randomized studies—of interventions (ROBINS-I) tool [22]. The following characteristics will be assessed: deviations from the intended intervention, missing outcome data, measurement of the outcome, selection of the reported result. Bias will be evaluated by two independent teams of two individuals. The alternating team will check the others work and settle any disagreements of individual judgements.

## Step 6: Extract pertinent data from final full-text articles and synthesize the information

The sixth step is to extract pertinent data from the included full-text articles and write a narrative synthesis. The following data will be extracted from study documents: participant characteristics of age, body mass, height, years of ultrarunning experience, finisher or non-finisher. Ultramarathon characteristics: distance of race, start time, elevation profile, environmental profile (temperature, humidity, windspeed, start elevation). Nutritional supplementation during the event: macromolecules (carbohydrates, fats, proteins), hydration (fluid type and pacing), and other supplements (vitamins and minerals). We will contact investigators for information that is not provided in the published document. We will report the average differences between finishers and non-finishers for macromolecules (carbohydrates, fats, proteins), hydration (fluid type and pace of ingestion), and other supplements (vitamins and minerals). Once all data are extracted, a narrative synthesis of the data will be written to report the main findings and implications of the systematic review.

## Step 7: Disseminate the findings of the systematic review

The final step is to disseminate the main findings and implications. The intention is to complete the systematic review by August 2022 and submit the narrative synthesis for publication in a peer-reviewed academic journal thereafter.

## Final remarks

To our knowledge, the proposed systematic review will be the first to describe the effects of nutrition and hydration between finishers and non-finishers of ultramarathon events as described by the scientific literature. Because of this, the review will fill an important gap in the literature. The protocol is presented here as a best practice [17] and to earn readers’ trust in the review protocol. Additionally, the procedures described here can provide others a framework to conduct their own systematic reviews if so desired.

## Supporting information

S1 Checklist PRISMA-P (Preferred Reporting Items for Systematic review and Meta-Analysis Protocols) 2015 checklist: Recommended items to address in a systematic review protocol*. (DOC)
